# Supplementary material for: Evaluating the Impact of Different Hypercaloric Diets on Weight Gain, Insulin Resistance, Glucose Intolerance, and its Comorbidities in Rats
Source: Nutrients. 2019 May 28;11(6):1197. doi: 10.3390/nu11061197 (PMC6627141; doi:10.3390/nu11061197)
Supplement: Supplementary file 1 [file nutrients-11-01197-s001.pdf]

Supplementary Data

Table S1 – Composition of 45% lipid-rich diet

**Composition**	
**Nutritional additives**	**Per kg**
Vitamin A	7600 IU
Vitamin D3	1900 IU
Iron	49.5 mg
Manganese	13 mg
Zinc	41.2 mg
Copper	7.4 mg
Iodine	0.26 mg
Seline	0.19 mg
Molybdenum	0.19 mg
**Preservatives**	
Potassium citrate	
**Colorants**	
Red Ponceau 4R	100 mg
**Analytical constituents**	**%**
Crude protein	21.00
Crude oils and fats	23.00
Crude fibres	4.50
Crude ash	3.50

Table S2– Composition of 60% lipid-rich diet

**Composition**	**%**
**Protein**	**23.1**
Arginine	0.90
Histidine	0.67
Isoleucine	1.24
Leucine	2.24
Lysine	1.88
Phenylalanine	1.24
Tyrosine	1.31
Threonine	1.0
Valine	1.47
Aspartic Acid	1.66
Glutamic Acid	5.28
Proline	3.04
Serine	1.43
**Fat**	**34.9**
Cholesterol	301 ppm
Linoleic Acid	4.70
Linolenic Acid	0.39
Arachidonic Acid	0.06
Omega-3 Fatty Acids	0.39

Total Saturated Fatty A	13.68
Total Monounsaturated Fatty Acids	14.00
Polyunsaturated Fatty Acids	5.15
**Fiber (max)**	**6.5**
**Carbohydrates**	**25.9**
**Energy**	**5.10 kcal/g**
**From:**	**%**
Protein	18.1
Fat	61.6
**Minerals**	
Calcium	0.79
Phosphorus	0.59
Potassium	0.77
Magnesium	0.07
Sodium	0.15
Chloride	0.25
Fluorine	1.2 ppm
Iron	65 ppm
Zinc	46 ppm
Manganese	76 ppm
**Vitamins**	
Vitamin A	5.2 IU/g
Vitamin D-3 (added)	1.3 IU/g
Vitamin E	67.2 IU/kg
Vitamin K	0.65 ppm
Thiamin	6.2 ppm
Riboflavin	8.7 ppm
Niacin	39 ppm
Pantothenic Acid	21 ppm

Table S3 – Composition of Purina 5008

Composition	%
**Protein**	**23.5**
Arginine	1.44
Histidine	0.58
Isoleucine	1.20
Leucine	1.87
Lysine	1.40
Phenylalanine	1.08
Tyrosine	0.66
Threonine	0.90
Valine	1.19

Aspartic Acid	2.60
Glutamic Acid	4.77
Proline	1.63
Serine	1.20
**Fat**	**7.5**
Cholesterol	208 ppm
Linoleic Acid	1.37
Linolenic Acid	0.09
Arachidonic Acid	0.01
Omega-3 Fatty Acids	0.29
Total Saturated Fatty A	2.51
Total Monounsaturated Fatty Acids	2.32
**Fiber (Crude)**	**3.8**
**Energy**	
Gross Energy	4.15 kcal/g
Physiological Fuel Value	3.50 kcal/g
Metabolizable energy	3.31 kcal/g
Gross Energy	4.15 kcal/g
**Minerals**	
Calcium	1.0
Phosphorus	0.65
Potassium	1.10
Magnesium	0.20
Sodium	0.28
Chlorine	0.48
Fluorine	19 ppm
Iron	230 ppm
Zinc	73 ppm
Manganese	71 ppm
**Vitamins**	
Vitamin A	15 IU/g
Vitamin D (added)	3.3 IU/g
Vitamin E	55 IU/kg
Vitamin K	3.2 ppm
Thiamin	16 ppm
Riboflavin	5.0 ppm
Niacin	109 ppm
Pantothenic Acid	15 ppm

Table S4 – Composition of Standard diet

Composition	%

**Proximate Analysis**	
Moisture	10.00
Crude Oil	4.16
Crude Protein	21.86
Crude Fibre	4.33
Ash	7.89
Nytrogen Free Extract	51.24
**Amino Acids**	
Arginine	1.39
Histidine	0.54
Isoleucine	0.96
Leucine	1.81
Lysine	1.30
Phenylalanine	1.20
Tyrosine	0.85
Alanine	0.27
Aspartic Acid	1.34
Glutamic Acid	4.30
Proline	1.53
Serine	0.98
**Fatty acids**	
Lauric	0.05
Myristic	0.17
Palmitic	0.37
Stearic	0.10
Myristoleic	0.01
Palmitoleic	0.09
Oleic	1.00
Linoleic	1.25
Linolenic	0.17
Arachidonic	0.12
**Fiber and Carbohydrates**	
Total Dietary Fibre	15.70
Pectin	1.47
Hemicellulose	9.33
Cellulose	4.04
Lignin	1.50
Starch	33.61
Sugar	5.84
**Energy**	
Gross Energy	15.10 MJ/kg
Digestible Energy	12.27 MJ/kg
Metabolizable energy	11.24 MJ/kg
Atwater Fuel Energy (AFE)	13.79 MJ/kg
AFE from Oil	11.35
AFE from Protein	26.51
AFE from Carbohydrate	62.13
**Minerals**	
Calcium	1.24
Phosphorus	0.80
Potassium	0.78
Magnesium	0.28
Sodium	0.24
Chloride	0.36
Fluorine	8.53 mg/kg
Iron	161.01 mg/kg

Zinc	46.90 mg/kg
Manganese	101.71 mg/kg
**Vitamins**	
Vitamin A	42522.66 IU/kg
Vitamin D-3 (added)	4369.41 IU/kg
Vitamin E	171.70 IU/kg
Vitamin K	40.72 mg/kg
Thiamin	49.77 mg/kg
Riboflavin	37.74 mg/kg

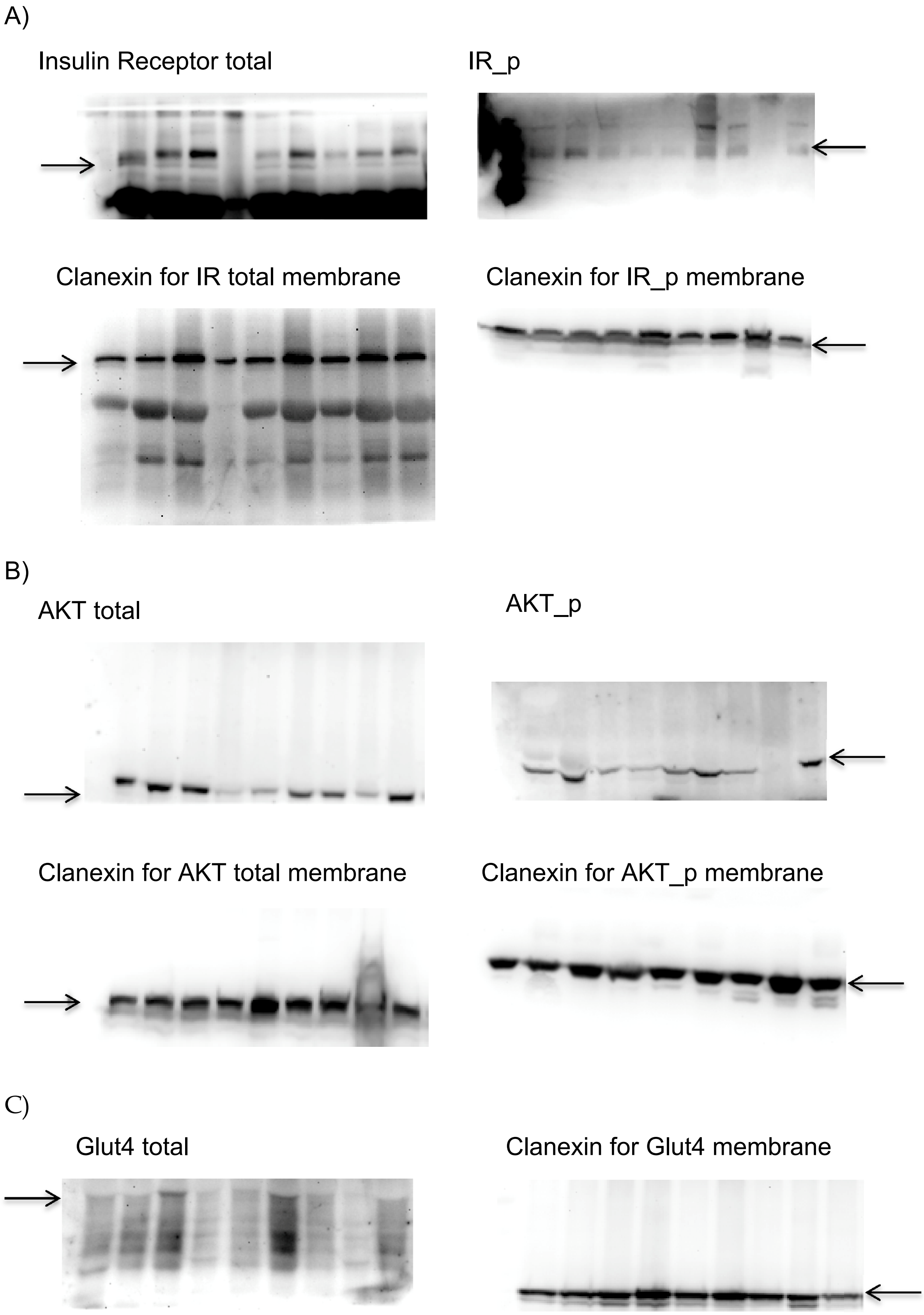

Figure S1- Membranes of the Western blots performed to study the alterations in the expression of proteins involved in insulin signaling in the adipose tissue, in animal models of obesity and type 2 diabetes. A) Western blot membranes for total insulin receptor (IR) protein (left panel) and its phosphorylated form (IR_p, right panel). Below shows

western blot membranes for the loading protein calnexin; B) shows western blot membranes protein kinase B, also known as AKT (left panel) and its phosphorylated form (AKT_p, right panel). Below shows western blot membranes for the loading protein calnexin; C) depicts western blot membranes for the expression of glut4 transporter and the respective loading protein calnexin. Black arrows point to the protein of interest.
